# Myricitrin Attenuates High Glucose-Induced Apoptosis through Activating Akt-Nrf2 Signaling in H9c2 Cardiomyocytes

**DOI:** 10.3390/molecules21070880

**Published:** 2016-07-05

**Authors:** Bin Zhang, Yaping Chen, Qiang Shen, Guiyan Liu, Jingxue Ye, Guibo Sun, Xiaobo Sun

**Affiliations:** 1 Institute of Medicinal Plant Development, Peking Union Medical College and Chinese Academy of Medical Sciences, Beijing 100193, China; zhangbin7@126.com (B.Z.); yejingxue2002@126.com (J.Y.); 2 Key Laboratory of Bioactive Substances and Resources Utilization of Chinese Herbal Medicine, Ministry of Education, Beijing 100193, China; 3 Beijing Key Laboratory of Innovative Drug Discovery of Traditional Chinese Medicine (Natural Medicine) and Translational Medicine, Beijing 100193, China; 4 Key Laboratory of Efficacy Evaluation of Chinese Medicine against Glyeolipid Metabolism Disorder Disease, State Administration of Traditional Chinese Medicine, Beijing 100193, China; 5 School of Life Science, Beijing Institute of Technology, Beijing 100081, China; Yp_chen@yeah.net (Y.C.); gyliu@bit.edu.cn (G.L.); 6 Center of Research and Development on Life Sciences and Environmental Sciences, Harbin University of Commerce, Harbin 150076, China; jinqilinsq@163.com

**Keywords:** hyperglycemia, oxidative stress, reactive oxygen species, apoptosis, H9c2 cell

## Abstract

Hyperglycemia, as well as diabetes mellitus, has been shown to trigger cardiac cell apoptosis. We have previously demonstrated that myricitrin prevents endothelial cell apoptosis. However, whether myricitrin can attenuate H9c2 cell apoptosis remains unknown. In this study, we established an experiment model in H9c2 cells exposed to high glucose. We tested the hypothesis that myricitrin may inhibit high glucose (HG)-induced cardiac cell apoptosis as determined by TUNEL staining. Furthermore, myricitrin promoted antioxidative enzyme production, suppressed high glucose-induced reactive oxygen species (ROS) production and decreased mitochondrial membrane potential (MMP) in H9c2 cells. This agent significantly inhibited apoptotic protein expression, activated Akt and facilitated the transcription of NF-E2-related factor 2 (Nrf2)-mediated protein (heme oxygenase-1 (HO-1) and quinone oxidoreductase 1 (NQO-1) expression as determined by Western blotting. Significantly, an Akt inhibitor (LY294002) or HO-1 inhibitor (ZnPP) not only inhibited myricitrin-induced HO-1/NQO-1 upregulation but also alleviated its anti-apoptotic effects. In summary, these observations demonstrate that myricitrin activates Nrf2-mediated anti-oxidant signaling and attenuates H9c2 cell apoptosis induced by high glucose via activation of Akt signaling.

## 1. Introduction

Diabetes mellitus (DM) is a chronic complex disease and is a leading cause of morbidity and mortality worldwide. The total number of diabetic patients is predicted to rise from 135 million in 1995 to 300 million in 2025 [1]. Diabetic cardiomyopathy (DCM) is characterized by left ventricular systolic and diastolic dysfunction independent of hypertension and coronary artery disease [2]. However, current studies on the pathogenesis of DCM remain incomplete.

Cardiomyocyte apoptosis is an important pathological alteration during DCM [3] and is thought to lead to persistent loss of effective myocardial contractile units [4], promote cardiac remodeling and eventually lead to cardiac failure. The apoptosis of cardiomyocytes can be induced by multiple factors, including oxidative stress [5], inflammation [6], endoplasmic reticulum (ER) stress [7] and advanced glycation end products (AGEs) [8]. Continuous high glucose in DM induces oxidative stress and excessive ROS production. Accumulating studies have reported that the excessive ROS can cause the increase of lactate dehydrogenase (LDH) leakage and malondialdehvde (MDA) level, and simultaneously inhibit some antioxidant enzymes including superoxide dismutase (SOD), catalase (CAT) and glutathione peroxidase (GSH-Px) [9]. The above oxidants and antioxidants have become the common indicators to reflect the oxidative stress level. Furthermore, the strategies to reduce oxidative stress-induced apoptosis remain limited. Thus, it is crucial to find the potent agent to protect cardiomyocyte against apoptosis caused by continuous hyperglycemia in DM.

Heme oxygenase-1 (HO-1), an endogenous cytoprotective enzyme with anti-apoptotic and anti-inflammatory properties, has recently attracted much attention [10]. It has been demonstrated that induction of HO-1 plays a crucial role in unconjugated bilirubin-mediated vascular benefits in diabetic mice [11]. In addition, studies have shown that the activation of nuclear erythroid-2-related factor (Nrf2) can promote HO-1 expression [12]. In contrast, deficiency of Nrf2 hinders HO-1 expression and aggravates oxidative stress both during myocardial hypertrophy induced by angiotensin II [13] and in hearts with transverse aortic constriction [14]. Under physiological conditions, Nrf2 is inactive in the cytoplasm, where it combines with its repressor, Keap1. Under conditions of oxidative stress, Nrf2 is released from the Keap1-Nrf2 complex and translocates to the nucleus [15]. A number of studies have demonstrated that activation of the phosphatidylinositol 3-kinase/protein kinase B (PI3K/Akt) signaling pathway can facilitate Nrf2 nuclear translocation [16]. Thus, activation of the PI3K/Akt pathway and the consequent modulation of HO-1 expression may be considered a possible approach to hinder hyperglycemia induced cell apoptosis.

**Figure 1:**
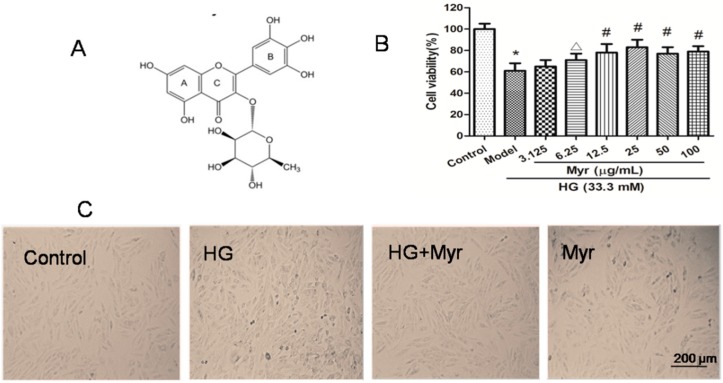
Structure of myricitrin and the effects of myricitrin on HG-induced H9c2 cell death and morphological changes. (**A**) The chemical structure of myricitrin; (**B**) Cell viability was assessed by the MTT assay. H9c2 cells were exposed to 33 mM glucose for 48 h in the presence or absence (Control) of myricitrin (25 μg/mL); (**C**) Representative photomicrographs of H9c2 cells in various experimental groups with or without high glucose treatment. The bar represents 200 μm. Values are represented as the mean ± SD (*n* = 9). The results were representative of three independent experiments. * *p* < 0.01 vs. control; ^Δ^ *p* < 0.05 vs. HG; ^#^ *p* < 0.01 vs. HG.

Myricitrin (Figure 1A) is a botanical flavone found in multiple herbal medicinal plants, such as *Myricacerifera*, *Myricaesculenta, Ampelopsis grossedentata* and other plants. Our laboratory previously demonstrated that myricitrin can attenuate endothelial cell apoptosis via PI3K/Akt signaling activation [17]. In addition, myricitrin reportedly possesses effective antioxidant, anti-inflammatory and antifibrotic activity [18]. Nevertheless, the potential role of myricitrin in diabetic cardiomyopathy remains unclear.

In the present study, we investigated the potential role of myricitrin against high glucose-induced H9c2 cell apoptosis and found that myricitrin inhibited H9c2 cell apoptosis induced by high glucose via Akt dependent Nrf2 signaling.

## 2. Results

### 2.1. Myricitrin Inhibits HG-Induced H9c2 Cell Death

As previously reported [4], the best condition for the establishment of a high glucose model was H9c2 cell treatment with 33.3 mM glucose for 48 h (Figure S1A3). Notably, myricitrin had no toxic impact on H9c2 cells (Figure S1B). Furthermore, the cells were pretreated with myricitrin for 4, 6 and 12 h at final concentrations of 3.125, 6.25, 12.5, 25, 50 and 100 μg/mL, and then with 33.3 mM glucose for an additional 48 h. Finally, the best condition was confirmed to establish the experimental model (33.3 mM for 48 h). The optimal concentration and pretreatment time with myricitrin was 25 μg/mL and 12 h, respectively (Figure 1B).

The morphological changes of H9c2 cells were monitored after HG (33.3 mM) treatment for 48 h. The H9c2 cells in the control group were fusiform with a full cytoplasm and clear edges (Figure 1C). In contrast, the HG group exhibited cell shrinkage (the morphological hallmark of apoptosis) and cell fragmentation with inconspicuous edges, features that were alleviated by myricitrin (25 μg/mL). Compared with the control group, a single treatment with myricitrin on the cells resulted in no significant difference in morphology.

### 2.2. Myricitrin Decreased Oxidants and Increased Antioxidants in H9c2 Cells Exposed to HG

**Figure 2:**
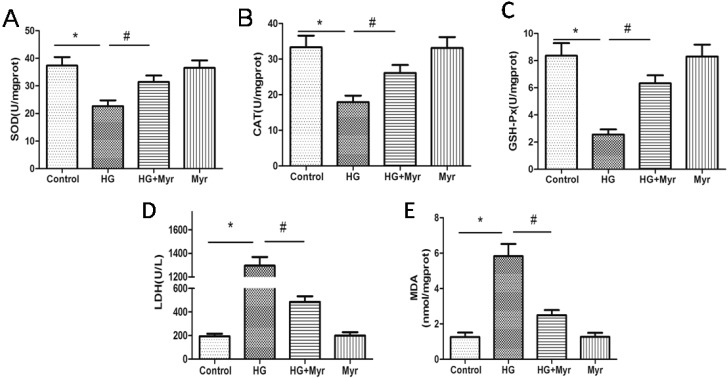
The effects of myricitrin on antioxidant enzyme activity in HG-induced H9c2 cells: SOD (**A**); CAT (**B**); GSH-Px (**C**); LDH (**D**); and MDA (**E**) activities were determined using the kits. Values are represented as the mean ± SD (*n* = 9). The results were representative of three independent experiments. * *p* < 0.01 vs. control; ^#^ *p* < 0.01 vs. HG.

Increasing evidence has shown hyperglycemia induces reactive oxygen species (ROS) with more oxidants and less antioxidants [19]. Thus, oxidant (LDH and MDA) and antioxidant levels (CAT, SOD and GSH-Px) were determined. Significantly, LDH leakage in the HG group increased in comparison with that in the control group (1295.89 U/L vs. 193.61 U/L, *p* < 0.01), which was markedly decreased by myricitrin treatment (Figure 2D). The results showed that the antioxidative enzymes SOD, CAT and GSH-Px were reduced in the HG group, suggesting that antioxidant capacity was compromised under high glucose conditions. Myricitrin enhanced antioxidative enzyme activities to protect H9c2 cells against HG injuries as indicated by the decreased MDA levels.

### 2.3. Myricitrin Suppresses HG-Induced Mitochondrial Superoxide (ROS) Production and Reduced MMP in H9c2 Cells

**Figure 3:**
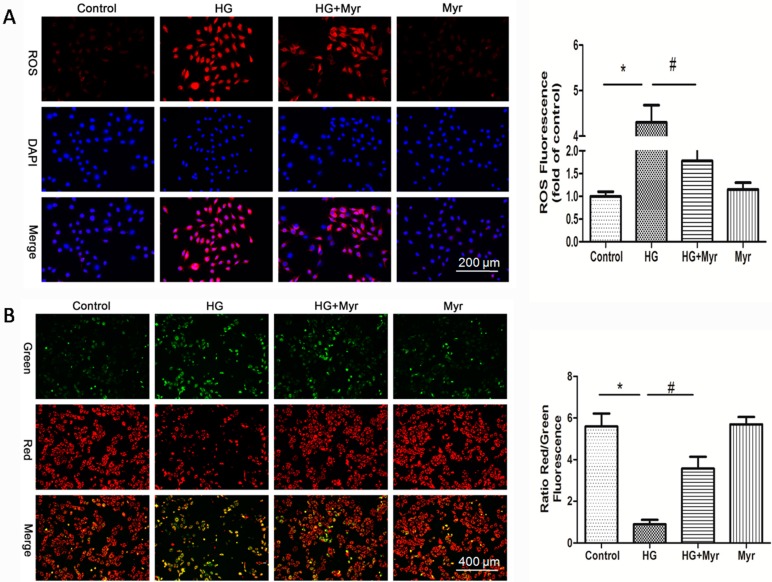
The effects of myricitrin on HG-induced ROS production and MMP (ΔΨ*m*) reduction in H9c2 cells. (**A**) Fluorescence images and bar diagram showed the ROS levels in the H9c2 cells; the fluorescence intensity of ROS was measured by a fluorescence microplate reader. The bar represents 200 μm; (**B**) Representative images and quantitative analysis of JC-1 staining. Treating H9c2 cells with HG caused a significant decrease in the in the ratio of red to green fluorescence intensity, which is a sign of the early stages of cell apoptosis. The bar represents 400 μm. Values are represented as the mean ± SD; *n* = 10 wells per group. * *p* < 0.01 vs. control; ^#^ *p* < 0.01 vs. HG.

A previous study demonstrated that HG increases the level of ROS leading to cell apoptosis [20]. Accordingly, we investigated ROS production in response to HG simulation and its regulation by myricitrin in H9c2 cells. Intracellular ROS exhibited red fluorescence under the microscope. In normal cells, few exhibited red fluorescence, indicating that the intracellular ROS level was low. The exposure to constant HG resulted in a remarkable increase in intracellular ROS generation (4.31-fold of the control group, *p* < 0.01), which could be significantly alleviated by pretreatment with myricitrin (1.78-fold of the control group, Figure 3A).

To investigate the effects of myricitrin on mitochondrial transmembrane potential (MTP), the JC-1 assay was used to assess mitochondrial depolarization. The mitochondria in normal H9c2 cells emitted red fluorescence after they were stained by JC-1. High glucose resulted in an increase in green fluorescence in H9c2 cells, indicating the depolarization of the mitochondrial transmembrane potential. Pretreatment with myricitrin restored MTP compared with MTP in cells exposed to HG (3.57 vs. 0.89, *p* < 0.01, Figure 3B).

### 2.4. Myricitrin Attenuates HG-Induced Cell Apoptosis in H9c2 Cells

**Figure 4:**
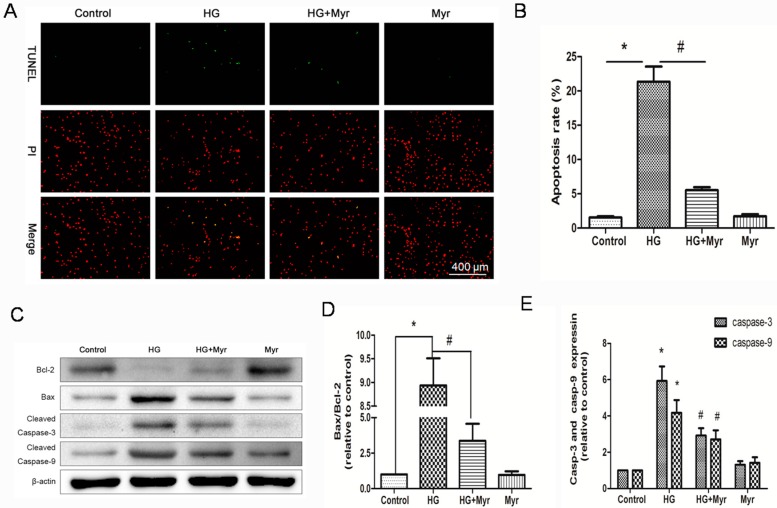
Protection effects of myricitrin against HG-induced H9c2 cells apoptosis. Pretreatment with myricitrin (25 μg/mL) for 24 h prior to HG, H9c2 cardiomyocytes apoptosis was assessed using TUNEL staining. (**A**) Representative images of TUNEL-positive nuclei in green fluorescent color and total nuclei staining with Propidium iodide (PI); (**B**) Bar diagram showing the relative proportion of TUNEL-positive cells (*n* = 5); (**C**) Western blot analysis of cleaved caspase-3, cleaved caspase-9, Bax and Bcl-2 expressions; (**D**) The ratio of Bax to Bcl-2 in various groups; (**E**) Cleaved caspase-3 and caspase-9 expressions in different groups. Values are represented as the mean ± SD (*n* = 3). * *p* < 0.01 vs. control; ^#^ *p* < 0.01 vs. HG.

To study the protective effect of myricitrin administration on HG-induced H9c2 cell apoptosis, TUNEL assay was performed. Cells with green nuclei were considered apoptotic. Few cells with nuclei staining green were observed in the control group (Figure 4A). After being exposed to HG for 48 h, approximately 22.13% of cells showed apoptotic hallmarks. Myricitrin significantly reduced the percentage of apoptotic cells to 5.54% (22.13% vs. 5.54%, *p* < 0.01, Figure 4B). The results from Hoechst33342/PI staining were accordance with TUNEL assay. About 55.11% H9c2 cells died after being exposed to high glucose (Figure S2B).

A western blot assay was performed to explore the molecular mechanism of myricitrin on the regulation of cell apoptosis. The results showed that pretreatment with myricitrin reversed the effects of HG by decreasing and increasing the expression levels of Bcl-2 and Bax, respectively (Figure 4C,D). Similarly, the expression levels of cleaved caspase-3 and caspase-9 were markedly suppressed by myricitrin pretreatment (Figure 4E). Thus, these results indicated that myricitrin could up-regulate anti-apoptotic proteins and down-regulate pro-apoptotic proteins.

### 2.5. Myricitrin Exerts Its Effects by Activating the PI3K/Akt Pathway and Nrf2/ARE Signaling

**Figure 5:**
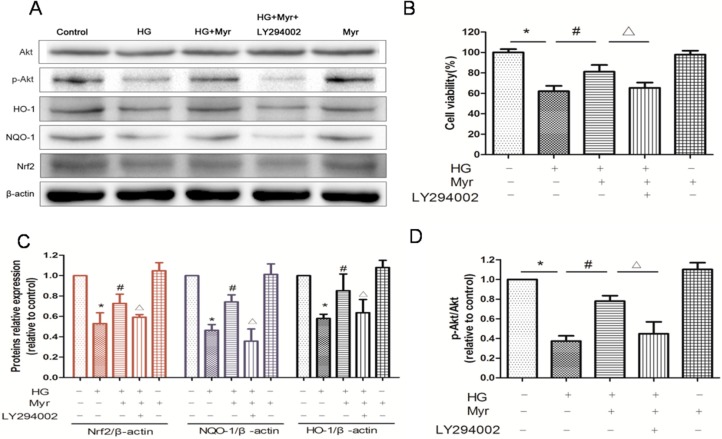
Myricitrin exerts its effects by activating the PI3K/Akt pathway and Nrf2/ARE signaling. (**A**) Representative immunoblots of the protein expression of phospho- and total Akt, HO-1, NQO-1 and Nrf2; (**B**) Cell viability in H9c2 cells with 4 h pretreatment of the PI3K inhibitor LY294002 (50 μM) was evaluated by MTT assay. *n* = 10 wells per group. Quantitative analysis of Nrf2, NQO-1 and HO-1 expression levels (**C**) and the statistical analysis of the p-Akt/Akt expression level relative to the control group (**D**). Values are represented as the mean ± SD (*n* = 3). * *p* < 0.01 vs. control; ^#^ *p* < 0.01 vs. HG; ^Δ^ *p* < 0.05 vs. HG + Myr. + represents treated; − represents untreated.

The antioxidant-responsive element (ARE) regulates the transcription of many anti-oxidant genes (i.e., heme oxygenase-1 (HO-1) and quinone oxidoreductase 1 (NQO-1)) and phase II detoxification enzymes [21]. Nrf2 regulates the transcriptional activation of HO-1 and NQO-1 by binding to ARE [22]. The above results showed that myricitrin inhibited the ROS production induced by HG. We thus investigated the impact of this agent on Nrf2 signaling. Western blot results showed that the expression of Nrf2 and Nrf2-mediated proteins (HO-1 and NQO-1) was obviously inhibited in H9c2 cells exposed to HG, the expression of which could be dramatically reversed by myricitrin pretreatment (Figure 5A,C).

**Figure 6:**
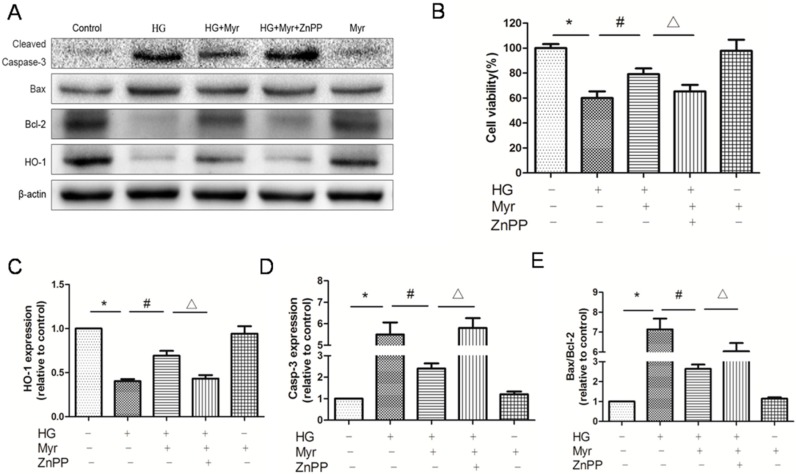
The anti-apoptotic effects of myricitrin were attenuated by HO-1 inhibitor. The expressions of target protein were evaluated by Western blot assay. (**A**) Representative immunoblots of the protein expression of HO-1, cleaved caspase-3, Bax and Bcl-2; (**B**) Cell viability of H9c2 cells with 48 h pretreatment of ZnPP (10 μM) was evaluated by MTT assay. *n* = 10 wells per group. Quantitative analysis of HO-1 (**C**) and cleaved caspase-3 expression levels (**D**) and the statistical analysis of the Bax/Bcl-2 expression level relative to the control group (**E**). Values are represented as the mean ± SD (*n* = 3). * *p* < 0.01 vs. control; ^#^ *p* < 0.01 vs. HG; ^Δ^ *p* < 0.05 vs. HG + Myr. + represents treated; − represents untreated.

The PI3K/Akt signaling pathway is involved in multiple cellular processes, including cell proliferation and survival. It was found that phosphorylation of Akt in H9c2 cells exposed to HG was decreased, which was restored by myricitrin pretreatment. The PI3K inhibitor LY294002 was used to further confirm the key role of the PI3K/Akt pathway in the anti-apoptotic effects of myricitrin pretreatment. Obviously, the up-regulation of phospho-Akt by myricitrin was abolished by LY294002 (Figure 5A,D) and the cell viability significantly decreased (Figure 5B). Furthermore, Nrf2, HO-1 and NQO-1 expression levels were correspondingly reversed. ZnPP, an HO-1 inhibitor, was used to explore the impact of myricitrin on HO-1 and apoptosis in the absence of HO-1. As we expected, after HO-1 inhibition, both the protective and anti-apoptotic effects of myricitrin on H9c2 cells under high glucose conditions were neutralized (Figure 6A,B). These results collectively indicate that myricitrin activates Nrf2-mediated anti-oxidant signaling and attenuates the H9c2 cell apoptosis induced by high glucose via activation of Akt signaling.

### 2.6 Discussion

In the current study, for the first time, we demonstrated that myricitrin attenuated HG-induced H9c2 cell apoptosis. In addition, myricitrin promoted antioxidative enzyme production. Moreover, myricitrin suppressed HG-induced mitochondrial ROS production and decreased MMP in H9c2 cells. Myricitrin inhibited HG-induced apoptotic protein expression, including caspase-3, caspase-9 and Bax. Further, it was confirmed that myricitrin not only activated Akt, but upregulated Nrf2-mediated HO-1 and NQO-1, which could be weakened by an Akt inhibitor (LY294002) and ZnPP. These observations collectively demonstrate that myricitrin activates Nrf2-mediated anti-oxidant signaling and attenuates HG-induced H9c2 cell apoptosis via activation of Akt signaling.

Evidence has accumulated that oxidative stress plays a key role in the pathogenesis of diabetes-induced cardiovascular disease [23,24], which is also closely related to the insulin resistance and impaired insulin secretion [25] that lead to the development of diabetic complications. Hyperglycemia has been reported to be associated with multiple factors that can result in oxidative stress, one of which is involved in AGEs [26]. It has been clarified that AGEs-induced ROS inhibits the translocation of Nrf2 into nucleus, which causes upregulation of antioxidant protein production [27]. In the development of diabetic nephropathy (DN), AGEs induces expressions of fibronectin and TGF-β1, which is attenuated by sirt1 through activating the Nrf2/ARE pathway [28]. One further trial has revealed that AGEs promotes ERK phosphorylation, resulting in a reduction in Nrf-2 and downstream pathway [29]. Thus, how to activate Nrf2-mediated signaling and antioxidant system remains meaningful for the treatment of diabetic complications. Numerous studies have demonstrated that the decrease in endogenous antioxidant capacity is tightly associated with the oxidative stress induced by hyperglycemia [30]. In the present study, we confirmed that consecutive HG not only decreased the H9c2 cell survival rate and impaired cell morphology but also significantly increased LDH leakage and the MDA level, while simultaneously severely hindering antioxidative enzyme activities including SOD, CAT and GSH-Px. However, pretreatment of H9c2 cells with myricitrin could reverse the decreased antioxidative enzymes, indicating that myricitrin retarded HG-induced oxidative stress. An accumulating body of evidence suggests that HG-induced oxidative stress leads to overproduction of ROS, which plays an essential role in the development of DCM [31]. Thus, we further detected the intracellular ROS level of H9c2 cells treated with HG. It was also confirmed that myricitrin treatment inhibited the increase in ROS content in H9c2 cardiomyocytes.

Numerous studies have demonstrated that oxidative stress is directly related to apoptosis in the hearts of diabetic patients or animals [32]. Given that myricitrin ameliorated HG-induced oxidative stress, we hypothesized that it subsequently inhibited apoptosis of cardiac myocytes. Metabolic disorders of the mitochondria are closely associated with apoptosis. In the current study, hyperglycemia caused loss of mitochondrial membrane potential (mtPTP opening), which was significantly restored by myricitrin. The apoptosis signaling pathways were further studied to explore the molecular basis of the effects of myricitrin. The results showed that cleaved caspase-3 and caspase-9 expression markedly increased in H9c2 cells treated with high glucose, suggesting the involvement of mitochondrial pathways. It was also found that myricitrin significantly inhibited cleaved caspase-3 and caspase-9 expression. Previous studies have reported that Bcl-2 family proteins are vital mediators of apoptotic processes [33]. Thus, Bax and Bcl-2 have a significant impact on mitochondrial permeability [34]. Additionally, there are studies demonstrating that overproduction of ROS increases the Bax/Bcl-2 expression ratio, which can decrease mtPTP and subsequently mediate caspase-3 expression [35,36]. In accordance with the previous studies, myricitrin pretreatment decreased the expression of pro-apoptotic Bax but increased the expression of anti-apoptotic Bcl-2. Collectively, myricitrin exerted its anti-apoptotic effect by eliminating ROS production and restoring MMP.

**Figure 7:**
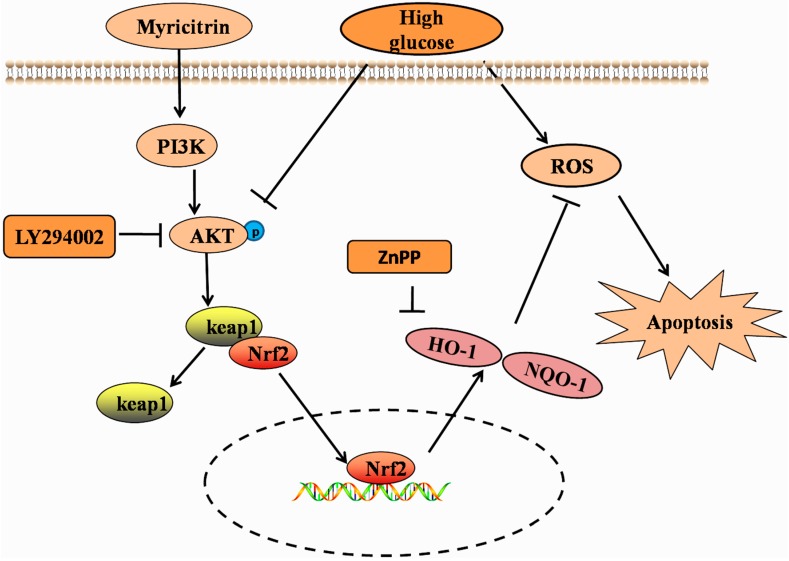
Schematic of the mechanism of myricitrin inhibition of HG-induced apoptosis via activation of PI3K/Akt signaling and up-regulation of HO-1 and NQO-1 in H9c2 cells.

Akt, as a classical signaling pathway, has been reported to be tightly related to cell apoptosis, the latter of which has been confirmed to regulate Nrf2 activation [18,21]. However, few studies have reported the impact of hyperglycemia on Akt signaling in H9c2 cells. Consistent with previous reports [16], our results showed that myricitrin increased HO-1 and NQO-1 expression, activated Akt phosphorylation and promoted Nrf2 signaling. Recent researches have reported that myricitrin can effectively inhibit protein kinase C pathways, which are mostly focus on antipsychotic effects of the agent [37,38]. It is highly limited to search for the related literature on myricitrin, as a PKC inhibitor to fight against apoptosis. Furthermore, our colleagues have found that myricitrin obviously promotes PI3K/Akt signaling pathway [17], which provides the solid foundation for our subsequent studies. To confirm whether myricitrin exerted its anti-apoptotic roles by Akt or other pathways, an Akt inhibitor (LY294002) was employed in this research. In expectation, the aforementioned anti-apoptotic effects of myricitrin were abolished by the addition of an Akt inhibitor (LY294002) or HO-1 inhibitor (ZnPP). The cell viability also decreased, suggesting that the anti-apoptotic effects of myricitrin were related to the activation of the PI3K/Akt pathway and HO-1 expression (Figure 7).

## 3. Experimental Section

### 3.1. Materials and Chemicals

All cell culture materials were obtained from GIBCO (Grand Island, NY, USA). 3-[4,5-Dimethylthylthiazol-2-yl]-2,5 diphenyltetrazolium bromide (MTT) was obtained from Sigma (St. Louis, MO, USA). All antibodies were obtained from Santa Cruz Biotechnology (Santa Cruz Biotechnology, Dallas, TX, USA), and other chemicals were purchased from Sigma.

### 3.2. Isolation and Purification of Myricitrin

Myricitrin was isolated from the ground bark of *Myriace rubrae* and was identified by Professor Rui-le Pan (Institute of Medicinal Plant Development, Beijing, China). The purity was over 99%, as detected by high-performance liquid chromatography (UV and DAD). The molecular structure of myricitrin is shown in Figure 1A.

### 3.3. H9c2 Cardiomyoblast Culture

Rat embryonic cardiomyoblast-derived H9c2 cells were obtained from the Cell Bank of the Chinese Academy of Sciences (Shanghai, China). Cells were cultured in Dulbecco’s modified Eagle’s medium (Gibco, glucose content, 5.5 mM) supplemented with 10% fetal bovine serum, 2 mM l-glutamine and a combination of penicillin and streptomycin (1%) in a humidified 5% CO_2_ atmosphere at 37 °C [4]. For all experiments, cells were plated at an appropriate density according to the experimental design and were grown for 24  h to reach 70%–80% confluence before experimentation.

### 3.4. Establishment of a High Glucose Model: Screening the Myricitrin Dosing Time and Concentration

To explore the optimal conditions for establishing this model, H9c2 cells were placed in 96-well plates at 10^4^ per well for 24 h. The cells were first treated for 24, 36, and 48 h with various concentrations of glucose. Mannitol (33.3 mM) was added to the cultures to rule out the effect of high osmolarity (Figure 1A). H9c2 cell viability was detected by the MTT method as described previously [39]. The MTT cell viability OD value was detected using a microplate reader (MQX 200, BioTek Instruments, Winooski, VT, USA). The cells were pretreated with myricitrin for 4, 6 and 12 h at final concentrations of 3.125, 6.25, 12.5, 25, 50 and 100 μg/mL and then with 33.3 mM glucose for an additional 48 h. The cell viability was measured as previously discussed. Finally, the best condition was confirmed in order to establish the experimental model (33.3 mM for 48 h). The optimal concentration and pretreatment time for myricitrin was 25 μg/mL and 12 h, respectively (Figure 1B).

Accordingly, cells were divided into the following groups: (1) control group (normal glucose concentration, 5.5 mM); (2) high glucose group (HG, 33.3 mM); (3) HG + myricitrin 25 μg/mL group; and (4) normal glucose + myricitrin 25 μg/mL group. The cells were pretreated with 25 μg/mL myricitrin for 12 h before incubation with 33.3 mM d-glucose for 48 h.

### 3.5. Measurement of Antioxidant and Oxidant Levels

The activities of lactate dehydrogenase (LDH), malondialdehyde (MDA), superoxide dismutase (SOD), catalase (CAT) and glutathione peroxidase (GSH-Px) were determined with the corresponding detection kit according to the manufacturer’s instructions (Nanjing Jiancheng Biotechnology Institute, Nanjing, China).

### 3.6. Terminal Deoxynucleotidyl Transferase-Mediated Dutp Nick End Labelling (TUNEL) Assay

Apoptotic H9c2 cells were visualized by TUNEL staining according to the manufacturer’s instructions (Biovision, Milpitas, CA, USA). Briefly, H9c2 cardiomyocytes were cultured in 24-well plates for 24 h. After treatment, the cells were fixed with 1% paraformaldehyde for 15 min. After twice washes with PBS, incubated the cells in the DNA labeling solution for 60 min at 37 °C, and then incubated the cells with anti-BrdU-FITC antibody solution in the dark for 30 min. Images were captured using a fluorescence microscope (DM4000B, Leica, Wetzlar, Germany), and the apoptotic cells were counted with at least 100 cells from five randomly selected fields in each group.

### 3.7. Hoechst33342/PI staining

In this study, cells were double-stained with Hoechst 33342 and propidium iodide (PI) for the qualitative analysis of cell deaths. H9c2 cardiomyocytes were cultured in 24-well plates for 24 h. After treatment, cells were washed twice with phosphate-buffered saline (PBS) and incubated with 10 μg/mL Hoechst 33342 (Sigma) dye for 15 min at 37 °C in the dark, and then 100 μg/mL PI (Sigma) was added. Stained nuclei were immediately observed by fluorescence microscopy. In normal cells, the nuclei appeared intact, and some were stained blue by Hoechst 33342, whereas cells with bright blue or red/pink nuclei were considered necrosis [40].

### 3.8. Measurement of Mitochondrial Superoxide (ROS)

MitoSOX Red (Molecular Probes), a mitochondrial superoxide indicator, was used to detect mitochondrial superoxide production in H9c2 cells. Briefly, after treatment, the cells were washed once with PBS and incubated with MitoSOX Red (5 μM) in the dark at 37 °C for 10 min. The cells were washed with PBS and then observed under a fluorescence microscope. The fluorescence of MitoSOX Red was detected on a microplate reader (Spectrafluor, TECAN, Sunrise, Austria) at the excitation and emission wavelengths of 510 nm and 580 nm, respectively.

### 3.9. Detection of Mitochondrial Membrane Potential (ΔΨm)

JC-1 (Invitrogen, Waltham, MA, USA) was employed to determine the changes in mitochondrial transmembrane potential (MMP). Briefly, H9c2 cells were incubated with JC-1 (5 μmol/L) at 37 °C for 30 min and were then washed with PBS followed by image acquisition using fluorescence microscopy.

### 3.10. Western Blotting Analysis

Western blots were performed as described previously [14]. Equal amounts (10 μg) of protein fractions were separated by electrophoresis on 10% SDS-PAGE, in which the protein samples were evenly loaded. The proteins were then transferred onto nitrocellulose membranes (Millipore Corporation, Bedford, MA, USA) in Tris-glycine buffer at 300 mA for 1 h. The membranes were blocked with 5% (*w*/*v*) nonfat milk powder in Tris-buffered saline containing 0.1% (*v*/*v*) Tween-20 (TBST) and then incubated overnight with appropriate primary antibodies at 4 °C. Afterwards, they were washed three times with TBST and incubated with secondary antibodies for 2 h at room temperature. The results were visualized by enhanced chemiluminescence.

### 3.11. Statistical Analysis

Data from at least three independent experiments were expressed as the means ± SD. Statistical comparisons between different groups were measured using one-way ANOVA followed by the Student–Newman–Keuls test. The level of significance was set at *p* < 0.05.

## 4. Conclusions

In summary, we herein demonstrate for the first time that myricitrin attenuates high glucose-induced H9c2 cell apoptosis by activating the Akt-dependent Nrf2 signaling pathway. Our study indicates that myricitrin may have potential therapeutic properties for the treatment of diabetic cardiomyopathy beyond blood glucose control.

## Supplementary Materials

Supplementary materials can be accessed at: http://www.mdpi.com/1420-3049/21/7/880/s1.
