# Supplementary material for: Myricitrin Attenuates High Glucose-Induced Apoptosis through Activating Akt-Nrf2 Signaling in H9c2 Cardiomyocytes
Source: Molecules. 2016 Jul 5;21(7):880. doi: 10.3390/molecules21070880 (PMC6274128; doi:10.3390/molecules21070880)
Supplement: Supplementary file 1 [file molecules-21-00880-s001.pdf]

Supplementary Materials: Myricitrin Attenuates High Glucose-Induced Apoptosis through Activating Akt-Nrf2 Signaling in H9c2 Cardiomyocytes

Bin Zhang, Yaping Chen, Qiang Shen, Guiyan Liu, Jingxue Ye, Guibo Sun and Xiaobo Sun

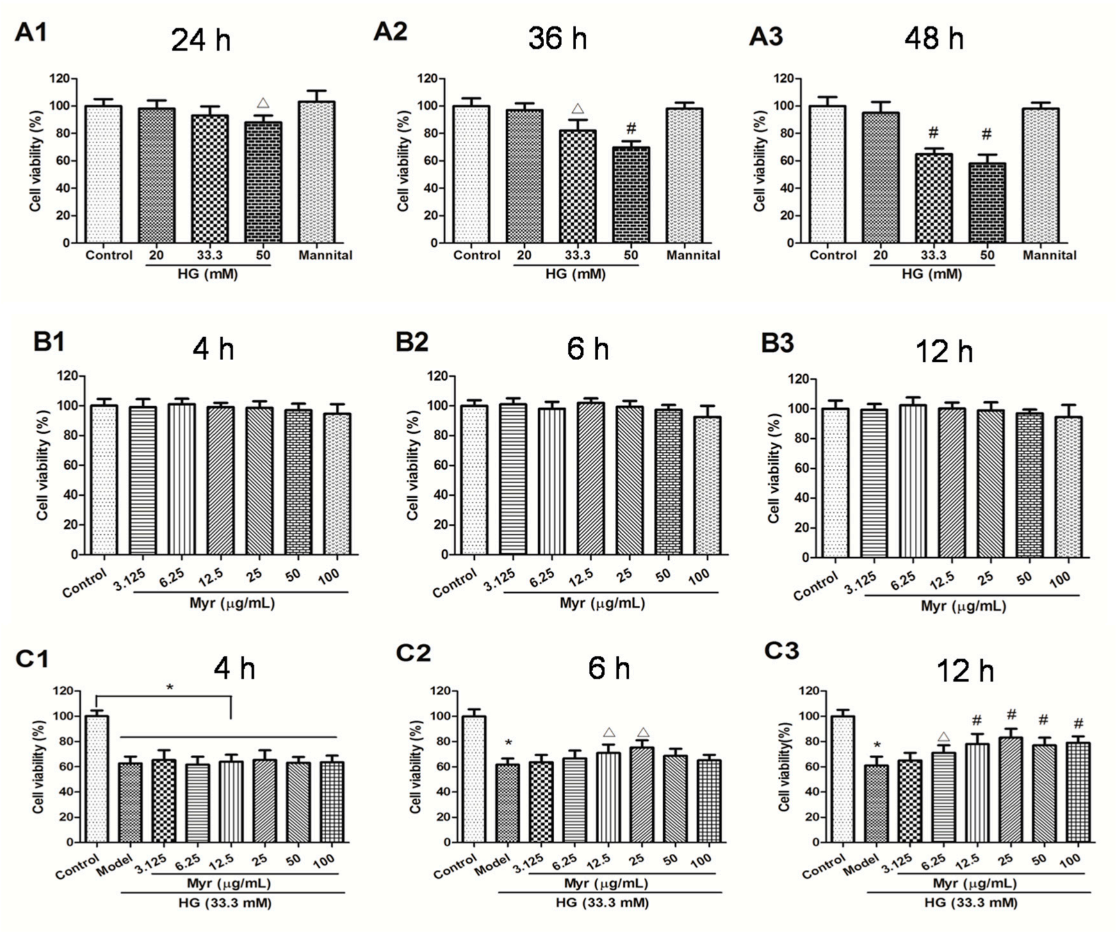

Figure S1. Exploration of the best conditions to establish a high-glucose model and pretreatment with myricitrin. (**A1**–**A3**) The viability of H9c2 cells exposed to various concentrations of high glucose (20, 33.3 and 50 mM) for 24, 36 and 48 h was determined by MTT assay. $^{\triangle}$ $p < 0.05$ vs. control; $^{\#}$ $p < 0.01$ vs. control; (**B1**–**B3**) The toxic effect of myricitrin on H9c2 cell viability was observed; (**C1**–**C3**) The protective effects of myricitrin on H9c2 cells exposed to high glucose (33.3 mM). * $p < 0.01$ vs. control; $^{\triangle}$ $p < 0.05$ vs. model; $^{\#}$ $p < 0.01$ vs. model. Values are represented as the mean ± SD; $n = 10$ wells per group.

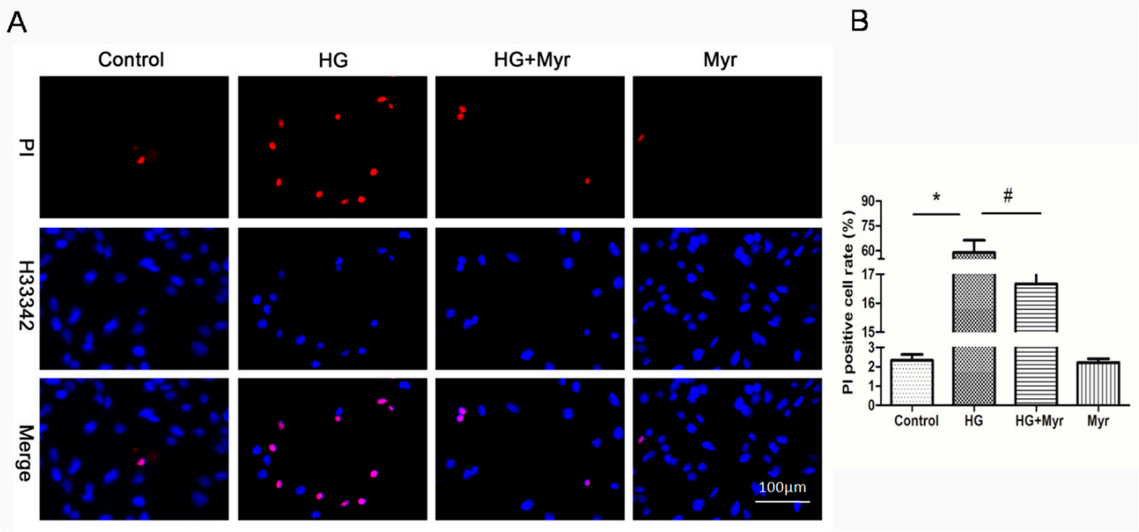

Figure S2. The protection effects of myricitrin on HG-induced H9c2 cell deaths determined by Hoechst 33342 staining. (**A**) Representative images of PI-positive nuclei in red fluorescent colour and total nuclei staining with Hoechst 33342. The bar represents 200 μm; (**B**) Bar diagram showing quantitative data of the PI positive rate (n = 5). * p < 0.01 vs. control; $^{\#}$ p < 0.01 vs. HG.
